# Dr. James Jay Mapes

**Published:** 1896-05

**Authors:** 


Dr. James Jay Mapes, of New York, died April 10, 1896, at Saranac Lake, after a prolonged illness. Dr. Mapes, who was one of the best-known among the younger physicians, was the son of Charles G. Mapes, the great-grandson of Gen. James Jonas Mapes, who commanded the American forces about New York during the war of 1812, and a nephew of Mary Mapes Dodge, editor of St. Nicholas. After taking his medical degree at the College of Physicians and Surgeons he served as interne at the New York hospital. During his medical course of study he spent his summers at the University of Edinburgh, where he won the first gold medal bestowed upon an American for highest honors in anatomy.

After graduation he went abroad again and continued his medical studies at Vienna and Paris. While in Paris he studied under Dr. Roux, at the Pasteur Institute, when the discovery of the antitoxin treatment for the cure of diphtheria was made, and when he returned home Dr. Roux gave him some vials of the antitoxin fluid, which was the first brought to this country. During his absence abroad Dr. Mapes was appointed resident physician of the Nursery and Child's Hospital in New York, and he took charge on January 1, 1895. At that time an epidemic of diphtheria prevailed at the hospital and the mortality was very large. Dr. Mapes applied the antitoxin treatment with marked success. He had written a number of articles on the subject.

